# Changes in muscle cell cation regulation and meat quality traits are associated with genetic selection for high body weight and meat yield in broiler chickens

**DOI:** 10.1186/1297-9686-41-8

**Published:** 2009-01-14

**Authors:** Dale A Sandercock, Zoe E Barker, Malcolm A Mitchell, Paul M Hocking

**Affiliations:** 1 Division of Genetics and Genomics, Roslin Institute and Royal (Dick) School of Veterinary Studies, University of Edinburgh, Roslin, Midlothian, Scotland EH25 9PS, UK; 2 Division of Cell Sciences, Faculty of Veterinary Medicine, University of Glasgow, Bearsden Road, Glasgow, G61 1QH; 3 Division of Farm Animal Science, Department of Clinical Veterinary Science, Langford House, Langford, Bristol, BS40 5DU, UK; 4 Sustainable Livestock Systems, SAC, Bush Estate, Penicuik, EH26 0PH, UK

## Abstract

Between-breed genetic variation for muscle and meat quality traits was determined at eight weeks of age in 34 lines of purebred commercial broiler and layer lines and traditional breeds (categories) of chickens. Between-breed genetic variation for plasma ion concentrations and element concentration in muscle dry matter and ash were determined. Plasma from broilers had higher concentrations of Na^+^, K^+^, Mg^++^, total and free Ca^++ ^and lower free:total Ca^++ ^than plasma from layer and traditional lines. Muscle from broilers contained more Na and higher concentrations of K, Mg and Ca per mg of ash but not of dry matter compared with layer and traditional lines. In comparison with layer and traditional lines, broiler genotypes were over three times heavier, their plasma creatine kinase activity (CK), a marker of muscle tissue damage, was higher, their breast muscle colour was lighter (L*) and less red (a*) and yellow (b*) in appearance, the initial and final pH of their muscles were lower, the pH change was higher and their breast muscle was more tender. Thus, genetic selection for broiler traits has markedly altered cation regulation in muscle cells and may be associated with changes in muscle cell function and the development of pathology and meat quality problems.

## Introduction

It is increasingly recognized that genetic selection for improved feed conversion efficiency, growth and muscle yields has resulted in alterations in ante- and post-mortem muscle status [1-4]. Low post-mortem muscle pH and associated pale meat and poor water holding capacity are particularly important because they affect the processing quality of meat [5]. These changes can be further influenced by factors such as heat, transport and handling stress [6-8]. Ante-mortem muscle problems have been identified by the measurement of plasma activities of intracellular enzymes such as creatine kinase (CK). Large increases of CK in the circulation indicate alterations in muscle membrane (sarcolemmal) permeability and therefore reflect muscle tissue damage [6,9,10]. Plasma CK activities increase with age and body size in lines of broiler chickens and turkeys selected for growth rate [4,11] and are consistent with histopathological evidence for muscle damage in both species [1,3].

Increased intracellular calcium concentrations are a central feature of irreversible cell damage [12,13]. Elevated intracellular calcium concentrations induced by calcium ionophores result in corresponding increases in plasma enzymes [14]. Proposed mechanisms of damage include calcium activation of phospholipase A_2 _activity and cellular proteases leading to membrane dysfunction [15]. Mitochondrial over-loading with calcium has also been proposed as a mechanism for muscle damage [14,16]. Further studies have demonstrated that the sodium ionophore monensin increases calcium entry into cells via sodium-calcium exchangers and increased CK efflux [17]. Increased muscle sodium and calcium concentrations and decreased muscle potassium and magnesium concentrations have been measured in needle muscle biopsies taken from Duchenne muscular dystrophy patients when compared with normal muscle [18]. Taken together, these experimental results suggest that disturbances in the regulation of cations other than calcium may also contribute to the aetiology of skeletal muscle damage, myopathy and ion channel dysfunction, and that the consequent disturbances in cation transport and distribution are the causative basis of many recognized disease states [19]. Muscle calcium, sodium, magnesium and potassium concentrations have never been reported in domestic fowl. Thus, the first objective of this study was to measure the muscle content of these cations in commercial broiler lines, which are highly selected for muscle growth and are susceptible to muscle damage, and in commercial layers and traditional breeds, which are unselected for muscle growth.

The second objective of the study was to determine the extent of genetic variation for meat quality traits in chickens that are potentially associated with changes in muscle cell function. We used a multi-strain experimental design to estimate the degree of genetic variation for a trait by determining the proportion of the total variation that is associated with different breeds or lines. Taylor [20,21] and Taylor and Hnizdo [22] have shown that a minimum of 25 lines with four unrelated individuals is close to the optimum for a range of objectives. Multi-strain experiments are therefore very efficient (provided that a large number of genetically distinct breeds or lines are available) and are useful to estimate the extent of genetic variation for traits that are difficult or expensive to measure.

## Methods

### Animals

Over 900 one-day old male chicks were obtained from 37 different pure lines consisting of 12 broiler (B), 12 layer (L) and 10 traditional breeds (T). The lines and breeds are listed in Table 1 with information on the source and mean body weight at eight weeks of age. The broiler and layer lines and traditional Brown Leghorn J line were sired by four males/line and the remaining traditional lines were the progeny of two males. The birds were randomly assigned to four large pens from three weeks of age where each pen contained at least one offspring from each sire. The birds were provided with *ad libitum *access to a commercial broiler starter diet from 0 to 35 days and a finisher diet from 36 to 64 days of age in six tubular feeders in each pen. The birds had unlimited access to water in suspended bell drinkers. A constant photoperiod of 16 h light and 8 h dark was maintained throughout the experiment. The experiment was conducted after ethical review and approval under relevant project and personal licences.

**Table 1:** Genetic lines (breed), classification (category), source and mean body weight at 8 weeks of age

**Breed**	**Category^1^**	**Source^2^**	**Body weight, kg**
**Auracana**	T	1	0.80
**Barnevelder**	T	1	0.82
**Brown Leghorn**	T	1	0.84
**Buff Orpington**	T	1	1.08
**Friesian Fowl**	T	1	0.63
**Ixworth**	T	2	1.56
**J line**	T	3	0.88
**Maran**	T	1	1.22
**White Dorking**	T	1	1.31
**White Sussex**	T	2	1.47
**Layer 1**	L	4	1.03
**Layer 2**	L	4	0.81
**Layer 3**	L	4	0.99
**Layer 4**	L	4	0.89
**Layer 5**	L	4	1.04
**Layer 6**	L	4	0.95
**Layer 7**	L	5	0.95
**Layer 8**	L	5	0.92
**Layer 9**	L	5	1.25
**Layer 10**	L	5	1.01
**Layer 11**	L	5	1.04
**Layer 12**	L	5	1.23
**Broiler 1**	B	6	3.79
**Broiler 2**	B	6	4.46
**Broiler 3**	B	6	4.36
**Broiler 4**	B	6	3.96
**Broiler 5**	B	7	3.50
**Broiler 6**	B	7	2.76
**Broiler 7**	B	7	3.66
**Broiler 8**	B	7	3.42
**Broiler 9**	B	8	4.02
**Broiler 10**	B	8	3.10
**Broiler 11**	B	8	4.30
**Broiler 12**	B	8	2.71

^1 ^T = traditional line; L = commercial layer line; B = commercial broiler line

^2 ^Origin of chicks; the same number indicates the same breeder of traditional breeds or commercial breeding company

### Sample collection

At eight weeks of age, one offspring of every sire was randomly removed from each pen, transferred to a holding pen and subjected to an overnight fast. The total number of birds available for the experiment was 136 from 12 B, 12 L and 10 T lines (Table 1). On the morning of the following day, a blood sample was obtained from each bird using a pre-heparinized needle and syringe and the birds were killed by intravenous injection of sodium pentobarbitone.

Blood samples were centrifuged for 5 min at 1500 × g and the plasma supernatant was frozen and stored at -20°C for later analysis. Plasma creatine kinase (CK) (EC 2.7.3.2) activity was determined using a commercially available kit (Alpha Laboratories) and total plasma Ca^++ ^and Mg^++ ^concentrations were measured using commercial diagnostic kits (Wako Chemicals GmbH) adapted for the use of a multi-well plate spectrophotometer (MR 5000, Dynatech laboratories, West Sussex, UK) as previously described by [2,6]. Plasma Na^+ ^and K^+ ^concentrations were measured using a 614 Na^+^/K^+ ^auto-analyser (CIBA-Corning Diagnostics Ltd). Free plasma Ca^++ ^concentrations were measured using a 634 pH/Ca^++ ^auto-analyzer (CIBA-Corning Diagnostics Ltd.)

### Meat quality determinations

Muscle samples (approximately 10 g) were removed from the left breast muscle for pH determination within 15 min of the bird's death (pH_i_) and 24 h post-killing and chilling (pH_u_). Samples were placed in self-sealed plastic bags, immediately frozen in liquid nitrogen and held at -80°C pending analysis to prevent post-mortem glycolysis [23]. Semi-frozen diced muscle samples were homogenised (1:10 wt/vol) in ice-chilled buffer (4°C) containing 5 mM sodium iodoacetate and 150 mMpotassium chloride (KCl) adjusted to pH 7.0 [24]. The pH was determined in the muscle homogenates using a combination pH electrode (Model FC200 Hanna Instruments, Leighton Buzzard, UK).

The carcasses were chilled for 24 h at 4°C and then both breast muscles (*m. pectoralis*) and both whole thighs (bone and muscles) were cut from the carcass and evaluated for muscle colour, lightness (L*), redness (a*) and yellowness (b*) using reflectance colorimetry (Minolta CR-300, CIELab, Minolta (UK) Limited, Milton Keynes, UK). Triplicate colour measurements were made on the ventral (anterior) aspects of both the breast (*m. pectoralis major*) and thigh muscles (*m. biceps femoris*).

After colour evaluation, blocks of muscle (approximately 50 g) were cut from the left pectoral muscle for shear force evaluation. Samples were placed in self-sealed plastic bags and immediately frozen in liquid nitrogen and held at -80°C pending analysis. The blocks of muscle were partly thawed on ice prior to cooking in plastic bags suspended in a water bath at 70°C for 35 min. From each cooked block, two sub-samples were obtained (3 × 1 × 1 cm; l × h × w) that were cut parallel to the muscle fibre axis. Peak force measurements (in triplicate) were taken along the length of each sub-sample using a materials force transducer (Model LRX, Lloyd Instruments, Hampshire, UK) fitted with a Warner-Bratzler shear blade.

### Muscle cation determination

The muscle samples were defrosted and cut into 2 g pieces and weighed to 0.0001 g (Sartorius Analytical AC1 210P). The muscle samples were placed in small polystyrene cartons and frozen at -20°C before being freeze-dried at -50°C (Super Modulyo, Edwards) to a constant weight (2–3 days). Freeze drying of samples is the recommended methodology for tissue cation determination as compared to "wet" methods, which exhibit lower accuracy and greater variability [25]. The freeze-dried samples were re-weighed for calculation of the tissue water content. The samples were transferred into pre-weighed 50 mL Pyrex beakers, placed in a cold muffle furnace and heated to 550 ± 5°C for 16 h (overnight). After this time, the beakers and their contents were removed from the furnace, placed in a desiccator and allowed to cool to ambient temperature. The beakers and contents were weighed to determine the ashed sample weight. Cations were extracted from the ashed samples with 10 mL of 6 N HCl. HCl was evaporated to dryness on a hot plate and then the residue was dissolved further in 10 mL of 6 N HCl. This solution was filtered through ashless filter paper (Whatman No. 1) into 100 mL volumetric flasks. The beaker was washed with no less than 50 mL of deionised water to ensure maximum cation extraction. The volume in the flask was made up to 100 mL with deionised water to provide the stock solution for the determination of magnesium, calcium, sodium and potassium. The stock solution was further diluted with deionised water, 1:50 and 1:150 respectively for sodium, magnesium and potassium. The stock solution for calcium determination was diluted 1:1 with LaCl_3 _(25 *mM*). Dilutions were carried out in 30 mL sterile sample tubes and stored in the freezer (-20°C) until all the batches were available for cation determination by atomic absorption spectrophotometry. All samples were analysed in an air-acetylene flame using appropriate wavelength, slit length and lamp currents. Each determination was obtained in duplicate and the mean of the two second readings for each sample was taken for analysis.

### Statistical analysis

The experiment was a randomised block design. Residuals were evaluated for normality and all analyses were conducted using the Residual Maximum Likelihood (REML) procedure of GENSTAT . Parameters of the statistical model were estimated by the marginal method of Breslow [26]. Variance components from a model with random effects for line, pen and residual were obtained. Between-breed genetic variation was defined as the intraclass correlation, *t*_*b *_= σ^2^_b_/(σ^2^_b _+ σ^2^_w_) where σ^2^_b _is the between and σ^2^_w _the residual (within line) component of variation. The analysis for each trait was repeated with a fixed effect for category (B, T and L) included in the model (*t*_*bw*_). Fixed effects were tested for significance by the method of Welham and Thompson [27]. Interactions between the fixed effects of category and tissue (*e.g*. breast *versus *thigh muscle) or at different times (*e.g*. pHi and pHu) were evaluated by comparing the deviance difference from omitting the interaction divided by the degrees of freedom against a χ^2 ^distribution.

The GENSTAT output provides an estimate of the standard error of σ^2^_b _and the statistical significance of the intraclass correlation was assessed as the ratio of the variance component to its standard error evaluated against a *t*-distribution. An approximate *a priori *average standard error of 0.1 was estimated from the formula for the variance of the intraclass correlation with breeds considered as a random effect from Taylor [[20], equation 4.4].

## Results

### Intra-class correlations and category differences

The magnitude of the intraclass correlations usefully standardizes the results for different traits but we have presented the importance of these results in terms of the significance of the between-breed variance component because this reflects more accurately the significance of genetic differences on trait variation. Between-category comparisons of broiler, layer and traditional lines are reported to dissect these variances into the relative contribution of genetic selection for broiler and egg traits on muscle quality.

#### Muscle composition

There was no detectable between-breed genetic variation in the gross composition of breast muscle. Breast muscle from B lines contained a similar amount of water as L and T lines but more organic (*p *< 0.05) and less inorganic (*p *< 0.001) matter (Table 2).

**Table 2:** Intraclass correlations for breast muscle composition and Na, Ca, Mg and K ion concentrations of breast muscle samples and plasma for 34 genetic lines of chickens calculated over all genetic lines (t_w_) and within category (t_wb_)

**Trait**	**Intraclass correlation^1^**		**Category average**			**SED^2^**
	**t_w_**	**t_wb_**	**Broiler**	**Layer**	**Traditional**	
**Breast muscle composition g/kg**						
Water	0.00	0.00	742	746	746	2.6
Organic matter	0.02	0.00	246	240	239	2.6*
Inorganic matter	0.14	0.00	12	14	15	0.5***
**Element concentration μg/mg breast muscle ash**						
Na	0.39^‡^	0.10	53.3	36.8	38.2	2.65***
K	0.25^†^	0.17	245	208	205	13.3**
Mg	0.22^†^	0.10	23.9	19.7	20.0	1.14***
Ca	0.24^†^	0.15	4.08	3.07	3.48	0.292**
**Element concentration μg/mg breast muscle DM**						
Na	0.24^†^	0.11	2.51	2.05	2.12	0.116***
K	0.08	0.09	11.6	11.4	11.2	0.44
Mg	0.18	0.18	1.14	1.08	1.10	0.041
Ca	0.09	0.07	0.194	0.172	0.185	0.0113
**Plasma ion concentration mmol/L**						
Na^+^	0.68^§^	0.28^†^	149.5	144.3	144.1	0.655***
K^+^	0.28†	0.04	4.44	3.67	3.81	0.129***
Mg^2+^	0.26†	0.14	0.979	0.963	0.853	0.0323***
Total Ca^2+^	0.63^§^	0.50^‡^	3.16	2.53	2.56	0.058***
Free Ca^2+^	0.33^‡^	0.28^†^	1.89	1.71	1.72	0.071*
Free/Total Ca^2+^	0.34^‡^	0.27^†^	0.60	0.68	0.68	0.029**

The means for broiler, layer and traditional lines of chickens are also presented

^1^Significance of between line (breed) variance component (*t*-test): † *p *< 0.05; ‡ *p *< 0.01; § *p *< 0.001

^2^Standard error of a difference between two category means: ** *p *< 0.05; *** *p *< 0.001

Intraclass correlations for the concentrations of Na, K, Mg and Ca in muscle ash and muscle dry matter were generally not statistically significant based on a test of the significance of the between-breed component of variance (Table 2). The concentrations of these elements expressed as a proportion of inorganic matter (ash) were all greater in B lines compared with L and T lines (*p *< 0.01) whereas only Na was higher (*p *< 0.001) when the results were expressed as a proportion of dry matter.

#### Plasma ion concentrations

Intra-class correlations for plasma ion concentrations were high for Na^+ ^and Ca^++ ^and were of marginal significance for K^+^, Mg^++^, free Ca^++ ^and the ratio of free:total Ca^++ ^(Table 2). The magnitude of the intraclass correlations for Na^+^, K^+ ^and Mg^++ ^were low when determined within categories, which contrasted with those for Ca^++ ^that were similar. Plasma from B lines contained more Na^+^, K^+^, total Ca^++ ^(*p *< 0.001) and free Ca^++^(*p *< 0.05) whereas the ratio of free:total Ca^++ ^was lower in B than L or T lines (*p *< 0.01). Plasma Mg^++ ^was similar in B and L lines and lower in T lines (*p *< 0.001).

#### Body weight and CK activity

Mean body weights at eight weeks for traditional, layer and broiler genotypes ranged respectively from 0.63 to 1.56, 0.81 to 1.25 and 2.71 to 4.46 (Table 3). The intra-class correlations for body weight and CK were very high and were substantially lower when calculated within categories (Table 3). The mean body weight of B lines was more than three times greater (*p *< 0.001) than that of T and L lines, which were similar. CK activity was approximately four times greater in B than in L and T lines (*p *< 0.001).

**Table 3:** Intraclass correlations for body weight, plasma creatine kinase activity, pH, tenderness, breast and thigh muscle colour at 8 weeks of age for 34 genetic lines of chickens calculated over all genetic lines (t_w_) and within category (t_wb_)

**Trait**	**Intraclass correlation^1^**		**Category average**			**SED^2^**
	**t_w_**	**t_wb_**	**Broiler**	**Layer**	**Traditional**	
**Weight and CK**						
Live weight, kg	0.96^§^	0.68^§^	3.67	1.01	1.06	0.168***
ln CK (iu/mL)	0.89^§^	0.62^§^	6.73 (835)	5.20 (181)	5.35 (211)	0.161***
**Breast muscle pH and toughness**						
pHi	0.34^‡^	0.35	6.09	6.16	6.16	0.017***
pHu	0.68^§^	0.51^‡^	5.69	5.87	5.81	0.034***
ΔpH	0.69^‡^	0.33^‡^	0.40	0.29	0.35	0.036**
Force N	0.58^‡^	0.40†	31.1	36.1	35.5	1.04***
**Breast muscle colour**						
Lightness (L*)	0.52^§^	0.50^‡^	55.0	53.6	53.5	0.83
Redness (a*)	0.49^‡^	0.14	2.95	5.74	5.21	0.372***
Yellowness (b*)	0.72^§^	0.35^‡^	2.14	5.41	3.77	0.364***
**Thigh muscle colour**						
Lightness (L*)	0.44^‡^	0.45^‡^	51.3	51.9	52.1	1.03
Redness (a*)	0.20†	0.20	6.55	6.24	7.03	0.541
Yellowness (b*)	0.62^§^	0.32^‡^	-1.34	1.45	0.92	0.411***

The means for broiler, layer and traditional lines of chickens are also presented

^1^Significance of between line (breed) variance component (*t*-test): † *p *< 0.05; ‡ *p *< 0.01; § *p *< 0.001

^2^Standard error of a difference between two category means: ** *p *< 0.05; *** *p *< 0.001

#### Muscle pH and toughness

The intra-class correlations for muscle pH immediately after killing (pHi) were similar overall and within categories and slightly lower in B lines compared with L and T lines (*p *< 0001). The intraclass correlations for muscle pH 24 h post-killing (pHu) and ΔpH were higher than pHi whereas only pHu was greater when calculated within categories (Table 3). A significant interaction (*p *< 0.001) occurred between categories and initial and ultimate pH caused by a larger decline in B than L and T lines. Initial and final pH were lower (*p *< 0.01) in B compared with L and T lines (sed within time = 0.023), which were similar.

The intraclass correlation for muscle toughness (Warner-Bratzler shear) was moderately high (0.58) and was decreased by one-third when calculated within categories (Table 3). The means for breast muscle from L and T lines were similar and indicated greater toughness than that from B lines (*p *< 0.001).

#### Breast and thigh meat colour

Intra-class correlations for muscle colour traits (Table 3) were similar overall and within categories for lightness (L*). Values of the intraclass correlation for redness (a*) were low and not significant for thigh muscle whereas those for breast muscle were high overall and low within categories. These results are consistent with a significantly lower a* value for breast but not thigh muscle in B lines compared with T and L lines. The results for yellowness (b*) in both muscles were similar and showed a large reduction in the intraclass correlation within categories compared with the overall value and significantly lower mean values in B lines compared with T and L lines (*p *< 0001).

Significant interactions (*p *< 0.01) occurred between category and breast or thigh for L*, a* and b*. L* was lower in thigh than in breast and the difference was much larger in B lines (sed between breast and thigh within category = 0.484). Redness values (a*) were similar in breast and thigh muscle for *L *and lower in breast compared with thigh muscle in T (*p *< 0.001; sed = 0.346); a* in breast muscle was lower in B lines compared with L and T lines and was similar in all three categories in thigh muscle (sed within site = 0.446). In thigh muscle, b* was lower in B lines compared with L and T lines, which were similar (sed within site = 0.349). However, in breast muscle, b* was lower in B than L lines and the T lines were intermediate (sed within category = 0.244).

### Between-breed genetic correlations

Between-breed correlations are presented in Additional file 1. Correlations based on all 34 genetic lines are below and correlations for the combined L and T lines only are above the diagonal. The most noticeable feature of the results is the presence of strong correlations between many traits and body weight and CK in the full data set that are weak and non-significant in the traditional and layer lines. There are also several strong correlations between thigh and breast colour, pHi, pHu, ΔpH and shear force that are not present among L and T lines. These data suggest that there is no relationship between pH and toughness in the lines that have not been selected for broiler traits compared with moderately strong positive correlations between pHi or pHu and toughness and a negative correlation with ΔpH in the full data set. There were weak, non-significant correlations between shear force and plasma ion concentrations in L and T lines whereas the relationships were negative and significant for Na^+^, total and free Ca^++^. High plasma ion concentrations were negatively and positively related respectively to breast muscle inorganic and organic matter concentrations in the full data set and were very weak and non-significant in the T and L lines. High plasma ion concentrations were positively related to element concentration in breast muscle ash (0.46 to 0.75) in the full data set but correlations were negative (-0.23 to -0.47) in the T and L lines. Correlations for total Ca^++ ^were similarly positive in the full data set (0.41 to 0.73) and were weak and not significant in the T and L lines (-0.16 to 0.14). Furthermore, positive correlations were found between Na and K, Mg and Ca in breast ash and DM (0.41 to 0.50) but not in the T and L lines (-0.19 to 0.13).

## Discussion

Previous studies in our laboratories have demonstrated that genetic selection for increased muscle mass in poultry is associated with an increased incidence of spontaneously occurring skeletal muscle abnormalities (idiopathic myopathy). The condition is characterised by degenerative histological changes such as hyaline (hypercontracted) fibres, fatty infiltration, fragmentation of the sarcoplasm, mononucleocyte infiltration and focal necrosis [1,2]. In addition, intensively selected poultry lines exhibit increases in plasma activity of the muscle enzyme creatine kinase (CK), which is released into the circulation as a consequence of muscle damage [4]. Previous investigations of the effect of genetic selection on idiopathic myopathy in poultry concern a small number of studies comparing small numbers of genetically divergent lines [1,2,4]. The prevalence and the extent of genetic variation for these genetic pathologies and their generality have not been determined.

Differences in muscle composition between the B lines and the L and T lines complicate the analysis of muscle cation contents. Historically, tissue cation contents have been expressed as a concentration per gram dry weight. However, our results show that the relative inorganic contents of tissues from the B lines were significantly lower than in the L and T lines. As a consequence, results expressed as an amount per unit of dry weight will differ from those expressed as a proportion of ash weight.

It is clear from the results of this experiment that B lines appear to exhibit consistently higher plasma cation concentrations than L and T lines and muscle cation concentrations are also higher in the B lines. The caveat is that this result is markedly affected by the unit weight calculation employed because B lines had significantly greater muscle organic than inorganic content. It could be argued that as broilers have a greater plasma sodium concentration than other categories, then the extra-cellular fluid (ECF) contribution to tissue sodium content [28] may account for the differences in this parameter between the broiler and layer or traditional genotypes. The contribution of plasma and interstitial fluid compartments to total tissue sodium content was estimated from the relative volumes of these tissue spaces for skeletal muscle taken from the literature [29-34] and multiplied by the plasma concentrations of sodium for each category (plasma and interstitial sodium concentrations being very similar). Based on the knowledge of total tissue water content and the calculated sodium concentrations in the extra-cellular compartments, it was possible to estimate the effect of sodium content in these tissue spaces on that in the intracellular compartment. The differences in the ECF contribution between the broiler genotypes and the other two categories were estimated as 3.6% and the corresponding difference for tissue sodium content was 21.0%. Therefore, it was concluded that the observed higher sodium concentration in tissue from broiler type birds was attributable to genuine differences in the relative intracellular sodium content. These results suggest that differences in cation regulation exist between B lines and other chicken lines. The higher concentration of Na^+ ^in broiler muscle compared with that in unselected lines is potentially important as alterations in muscle cation homeostasis may underlie the initiation of muscle degeneration [17] and subsequent reductions in meat quality. Furthermore in man, raised skeletal muscle sodium content is associated with injury and disease states [35].

The results in Table 3 show that there is significant genetic variation for commercially important muscle and meat quality traits and that genetic selection could be used to improve muscle pH and meat toughness (Table 3). This is consistent with recent estimates of genetic parameters and detection of quantitative trait loci for meat quality traits in a number of genetic lines [36-39]. Low muscle pH and higher pH decline post-slaughter are associated with decreased water holding capacity and increased keeping quality (resistance to microbial development). It is interesting that muscles from broilers were more tender than from layer lines and traditional breeds, a result that was confirmed by taste panel assessments (unpublished results).

Category comparisons show that breast muscle from broilers is lighter in colour and less red and yellow in its composition than tissue from L and T lines as expected but the intraclass correlations are moderate in size even within categories. Variation in the colour of breast muscle filets is commercially important and significant differences among lines of broilers suggest that genetic selection could be effective in decreasing this variability. The greater yellow colour in the breast muscle in L and T lines may be a result of greater fatness in these groups compared with B lines or there may be differences in the colour of fat due to genetic differences in carotene deposition [40].

Some caution is warranted in interpreting the between-breed genetic and phenotypic correlations in Additional file 1 as the data set is not large *i.e*. it consists of 34 and 22 data points respectively for the full and reduced data sets and there are nearly 400 correlations for each. Furthermore, the range of body weights in B lines was high compared with that in T and L lines (Table 1). However, there are over 200 nominally significant between-breed genetic correlations but less than 50 phenotypic correlations compared with an expected error of 20 at *p *< 0.05. Nevertheless, taking a cautious approach, the data suggest that the quality of the breast muscle of heavy broiler genotypes has been changed in relation to unselected birds and is consistent with conclusions based on comparisons of two to four stocks that the cell membranes of muscle tissues in broiler lines are functionally different compared with those in unselected lines [4]. Correlations for muscle Na^+ ^content and plasma CK activity are consistent with the *in vitro *studies on muscle damage in chicken muscle by Sandercock and Mitchell [17]. The results are useful for developing hypotheses that carry more weight because of the multi-strain experimental design than single line comparisons. The data suggest that raised Na^+ ^and Ca^++ ^in muscle of broilers, even after allowing for the high plasma concentrations, may underlie the link between ante-mortem muscle pH (glycolysis), rate of pH decline after death and muscle proteolysis, fibre fragmentation and reduced water holding capacity. These changes are probably the result of the high metabolic demand and mass of breast muscle tissue and contribute to muscle damage and changes in meat quality [1,2]. These results also suggest molecular mechanisms that may provide opportunities in studies aimed at improving muscle quality by genetic means.

## Conclusion

Genetic selection for broiler traits has markedly altered inter-compartmental cation regulation in muscle cells of current meat type birds, which reflects adaptive responses to high tissue metabolic demands. Altered intracellular cation distributions may contribute to changes in muscle cell function in rapidly growing meat birds and in turn mediate the development of muscle pathologies and meat quality problems. The imposition of stress upon broiler birds further exacerbates these problems and underlies additional product quality decrements and the development of muscle pathologies. Changes in calcium and other intracellular cation homeostasis may therefore represent the mechanisms of both growth and stress induced alterations in muscle and meat quality attributes in chickens. In contrast to genetic selection for meat characteristics, selection for high rates of egg laying has not affected muscle function at eight weeks of age compared with traditional breeds of chickens.

## Competing interests

The authors declare that they have no competing interests.

## Authors' contributions

DAS conducted the experiment, collated the data and supervised the analysis of ions and elements. ZB performed the ion and element analyses. MAM advised on the collection and interpretation of the data. PMH developed the experimental design and obtained funding, assisted in data collection, and analysed the data. DAS and PMH wrote the draft manuscript. All authors read and approved the final manuscript.

## Supplementary Material

Additional file 1 **Table S1. **Between-breed genetic correlations between muscle and meat quality traits at eight weeks of age in 34 broiler layer and traditional lines of chickens. Click here for file
