# Supplementary material for: Changes in muscle cell cation regulation and meat quality traits are associated with genetic selection for high body weight and meat yield in broiler chickens
Source: Genet Sel Evol. 2009 Jan 14;41(1):8. doi: 10.1186/1297-9686-41-8 (PMC2637030; doi:10.1186/1297-9686-41-8)
Supplement: Additional file 1 — Table S1. Between-breed genetic correlations between muscle and meat quality traits at eight weeks of age in 34 broiler layer and traditional lines of chickens. [file 1297-9686-41-8-S1.doc]

**Table S1:** **Between-breed genetic correlations between muscle and meat quality traits at eight weeks of age in 34 broiler layer and traditional lines of chickens.**

| Trait | LW | CK | Thigh colour | | | Breast colour | | | Breast muscle | | | | | | | Plasma concentration, mmol/L | | | | | | Breast muscle, µg/mg ash | | | | Breast muscle, µg/mg DM | | | |
| --- | --- | --- | --- | --- | --- | --- | --- | --- | --- | --- | --- | --- | --- | --- | --- | --- | --- | --- | --- | --- | --- | --- | --- | --- | --- | --- | --- | --- | --- |
|  |  |  | a* | b* | L | a* | b* | L | pHi | pHu | ΔpH | F N | H_2_O | Org | Inor | Na^+^ | K^+^ | Mg^+^ | TCa | FCa | F/T Ca | Na | K | Mg | Ca | Na | K | Mg | Ca |
| Live weight, g |  | 0.41 | 0.02 | -0.40 | -0.17 | -0.02 | -0.31 | -0.16 | -0.06 | -0.16 | 0.11 | -0.07 | -0.13 | 0.13 | -0.02 | 0.18 | 0.05 | -0.21 | -0.05 | 0.29 | 0.30 | -0.09 | 0.23 | 0.08 | 0.18 | -0.12 | 0.24 | 0.04 | 0.19 |
| CK^1^ ln(IU/L) | **0.96** |  | -0.08 | 0.12 | -0.03 | **-0.51** | -0.37 | 0.01 | 0.19 | -0.28 | 0.31 | -0.22 | 0.06 | -0.08 | 0.09 | 0.25 | 0.24 | -0.15 | 0.19 | 0.10 | 0.02 | -0.27 | -0.04 | -0.25 | -0.24 | -0.19 | 0.05 | -0.25 | -0.24 |
| Thigh colour (a*) | -0.04 | -0.03 |  | -0.01 | -0.38 | 0.09 | -0.12 | -0.23 | -0.17 | 0.09 | -0.14 | -0.01 | -0.16 | 0.16 | -0.02 | 0.14 | 0.29 | 0.04 | -0.12 | 0.10 | 0.15 | 0.17 | -0.09 | 0.04 | 0.25 | 0.08 | -0.21 | -0.07 | 0.20 |
| Thigh colour (b*) | **-0.78** | **-0.74** | 0.02 |  | 0.18 | -0.38 | 0.32 | 0.21 | 0.28 | 0.06 | 0.06 | -0.09 | -0.19 | 0.13 | 0.30 | 0.16 | 0.29 | 0.19 | 0.08 | 0.20 | 0.12 | -0.34 | -0.37 | **-0.44** | -0.38 | -0.27 | -0.41 | -0.51 | -0.41 |
| Thigh colour (L) | -0.12 | -0.18 | **-0.49** | 0.17 |  | -0.21 | -0.10 | **0.62** | 0.17 | -0.31 | 0.33 | -0.18 | 0.36 | -0.36 | 0.04 | -0.03 | 0.21 | -0.19 | 0.07 | 0.16 | 0.10 | -0.41 | -0.15 | -0.25 | -0.13 | -0.33 | -0.12 | -0.25 | -0.12 |
| Breast colour (a*) | **-0.85** | **-0.87** | 0.15 | **0.56** | 0.05 |  | 0.19 | -0.26 | -0.41 | 0.32 | **-0.43** | 0.30 | -0.08 | 0.10 | -0.11 | 0.12 | -0.25 | 0.13 | -0.27 | -0.30 | -0.12 | **0.44** | 0.05 | 0.17 | 0.28 | 0.40 | -0.07 | 0.14 | 0.36 |
| Breast colour (b*) | **-0.75** | **-0.77** | -0.08 | **0.72** | 0.04 | **0.65** |  | 0.31 | 0.05 | 0.28 | -0.22 | 0.11 | -0.24 | 0.23 | 0.08 | 0.25 | -0.06 | **0.49** | -0.35 | -0.19 | -0.03 | -0.20 | 0.00 | -0.04 | -0.24 | -0.22 | -0.02 | -0.07 | -0.24 |
| Breast colour (L) | 0.31 | 0.32 | -0.17 | -0.15 | 0.33 | **-0.39** | -0.03 |  | 0.23 | -0.13 | 0.20 | -0.17 | 0.13 | -0.12 | -0.05 | 0.29 | 0.32 | -0.12 | -0.24 | 0.13 | 0.21 | -0.39 | -0.13 | -0.19 | -0.11 | **-0.47** | -0.26 | -0.35 | -0.26 |
| pHi | **-0.66** | **-0.67** | -0.03 | **0.54** | 0.30 | **0.49** | **0.43** | -0.13 |  | -0.20 | **0.55** | 0.02 | 0.13 | -0.22 | **0.47** | 0.23 | 0.39 | -0.26 | 0.38 | 0.03 | -0.14 | -0.40 | **-0.43** | **-0.63** | -0.31 | -0.14 | -0.30 | **-0.55** | -0.28 |
| pHu | **-0.68** | **-0.73** | 0.10 | **0.56** | 0.04 | **0.73** | **0.56** | **-0.36** | **0.49** |  | **-0.93** | 0.16 | -0.27 | 0.24 | 0.12 | -0.10 | **-0.45** | **0.51** | -0.16 | **-0.48** | -0.37 | 0.18 | -0.20 | 0.00 | -0.20 | 0.20 | -0.25 | 0.01 | -0.21 |
| ΔpH | **0.42** | **0.47** | -0.13 | **-0.35** | 0.12 | **-0.58** | **-0.41** | **0.34** | -0.02 | **-0.88** |  | -0.13 | 0.28 | -0.29 | 0.07 | 0.17 | **0.53** | **-0.54** | 0.28 | 0.42 | 0.26 | -0.31 | 0.00 | -0.24 | 0.05 | -0.23 | 0.10 | -0.22 | 0.07 |
| Force N | **-0.69** | **-0.69** | -0.05 | **0.48** | 0.15 | **0.71** | **0.50** | -0.18 | **0.63** | **0.62** | **-0.37** |  | 0.17 | -0.19 | 0.16 | -0.20 | -0.20 | -0.08 | -0.12 | 0.07 | 0.10 | 0.15 | -0.09 | -0.08 | -0.08 | 0.25 | 0.03 | 0.02 | -0.13 |
| Water g/kg | -0.32 | -0.31 | -0.29 | 0.23 | **0.42** | 0.25 | 0.17 | 0.00 | 0.19 | 0.11 | -0.02 | 0.27 |  | **-0.98** | 0.03 | -0.24 | -0.05 | 0.00 | 0.30 | -0.16 | -0.27 | 0.02 | -0.18 | -0.07 | -0.31 | 0.30 | 0.04 | 0.21 | -0.35 |
| Organic matter g/kg | **0.45** | **0.44** | 0.27 | **-0.35** | **-0.44** | **-0.36** | -0.27 | 0.06 | -0.33 | -0.21 | 0.06 | **-0.35** | **-0.98** |  | -0.21 | 0.18 | 0.03 | -0.01 | -0.30 | 0.16 | 0.28 | 0.05 | 0.33 | 0.22 | 0.42 | -0.32 | 0.05 | -0.10 | 0.41 |
| Inorganic matter g/kg | **-0.70** | **-0.69** | 0.03 | **0.62** | 0.22 | **0.58** | **0.51** | -0.30 | **0.69** | **0.51** | -0.21 | **0.47** | 0.21 | **-0.42** |  | 0.30 | 0.14 | 0.08 | 0.01 | -0.03 | -0.07 | -0.39 | **-0.83** | **-0.84** | **-0.62** | 0.17 | **-0.48** | **-0.53** | -0.38 |
| Na^+^ mmol/L | **0.88** | **0.86** | 0.12 | **-0.66** | -0.22 | **-0.74** | **-0.66** | 0.29 | **-0.53** | **-0.59** | **0.39** | **-0.74** | **-0.41** | **0.50** | **-0.56** |  | **0.51** | 0.19 | -0.23 | 0.01 | 0.11 | -0.38 | **-0.45** | **-0.47** | -0.23 | -0.31 | **-0.57** | **-0.59** | -0.17 |
| K^+^ mmol/L | **0.68** | **0.67** | 0.14 | **-0.54** | 0.08 | **-0.56** | **-0.62** | **0.38** | -0.18 | **-0.51** | **0.48** | -0.31 | -0.28 | **0.35** | **-0.42** | **0.68** |  | -0.25 | -0.08 | **0.43** | **0.44** | -0.35 | -0.25 | **-0.43** | 0.05 | -0.39 | -0.39 | **-0.63** | 0.04 |
| Mg^+^ mmol/L | 0.29 | 0.26 | 0.13 | -0.26 | -0.20 | -0.18 | -0.06 | 0.02 | -0.19 | 0.10 | -0.22 | -0.15 | -0.23 | 0.25 | -0.19 | **0.36** | 0.31 |  | 0.03 | -0.29 | -0.28 | -0.21 | -0.12 | 0.00 | **-0.43** | -0.14 | -0.04 | 0.09 | -0.38 |
| Total Ca^2+^ mmo/L | **0.85** | **0.90** | -0.04 | **-0.73** | -0.22 | **-0.79** | **-0.74** | 0.26 | **-0.55** | **-0.70** | **0.50** | **-0.63** | -0.28 | **0.40** | **-0.64** | **0.78** | **0.62** | **0.37** |  | -0.02 | **-0.44** | 0.14 | 0.03 | -0.01 | -0.16 | 0.22 | 0.17 | 0.10 | -0.18 |
| Free Ca^2+^ mmol/L | **0.44** | **0.44** | 0.23 | -0.31 | -0.12 | **-0.46** | **-0.46** | 0.19 | -0.25 | **-0.56** | **0.51** | **-0.41** | **-0.34** | **0.38** | -0.28 | **0.50** | **0.57** | 0.08 | **0.44** |  | **0.90** | -0.34 | 0.14 | -0.15 | 0.25 | **-0.45** | 0.08 | -0.34 | 0.19 |
| Free/total Ca^2+^ | **-0.44** | **-0.48** | 0.23 | **0.43** | 0.10 | **0.37** | 0.33 | -0.08 | 0.30 | 0.17 | -0.03 | 0.27 | -0.04 | -0.04 | **0.35** | -0.33 | -0.11 | -0.31 | **-0.59** | **0.46** |  | -0.35 | 0.14 | -0.11 | 0.33 | **-0.50** | 0.02 | -0.34 | 0.29 |
| Na µg/mg ash | **0.81** | **0.76** | 0.07 | **-0.69** | -0.24 | **-0.64** | **-0.63** | 0.13 | **-0.62** | **-0.45** | 0.18 | **-0.60** | -0.27 | **0.42** | **-0.75** | **0.75** | **0.48** | 0.26 | **0.73** | 0.27 | **-0.48** |  | 0.29 | **0.59** | 0.39 | **0.79** | 0.12 | **0.56** | 0.31 |
| K µg/mg ash | **0.59** | **0.53** | 0.05 | **-0.56** | -0.31 | **-0.51** | **-0.37** | 0.11 | **-0.48** | **-0.41** | 0.21 | **-0.42** | **-0.37** | **0.53** | **-0.83** | **0.49** | 0.27 | 0.22 | **0.49** | 0.32 | -0.17 | **0.70** |  | **0.84** | **0.64** | -0.19 | **0.85** | **0.63** | **0.49** |
| Mg µg/mg ash | **0.61** | **0.58** | 0.07 | **-0.61** | -0.33 | **-0.46** | **-0.42** | 0.12 | **-0.72** | **-0.38** | 0.05 | **-0.40** | -0.25 | **0.43** | **-0.90** | **0.46** | 0.24 | 0.19 | **0.53** | 0.16 | **-0.37** | **0.74** | **0.81** |  | **0.55** | 0.13 | **0.63** | **0.86** | 0.39 |
| Ca µg/mg ash | -0.03 | **0.45** | 0.15 | **-0.57** | -0.13 | **-0.35** | **-0.50** | 0.06 | **-0.48** | **-0.39** | 0.19 | **-0.48** | **-0.39** | **0.51** | **-0.67** | **0.52** | **0.38** | -0.03 | **0.41** | **0.43** | -0.00 | **0.64** | **0.67** | **0.65** |  | -0.04 | 0.35 | 0.24 | **0.92** |
| Na µg/mg DM | **0.64** | **0.59** | 0.04 | **-0.58** | -0.13 | **-0.47** | **-0.56** | -0.06 | **-0.45** | -0.31 | 0.11 | **-0.52** | -0.06 | 0.15 | **-0.42** | **0.61** | **0.34** | 0.19 | **0.58** | 0.14 | **-0.48** | **0.89** | **0.41** | **0.50** | **0.45** |  | -0.07 | 0.38 | 0.04 |
| K µg/mg DM | 0.25 | 0.17 | 0.01 | **-0.34** | -0.16 | -0.24 | -0.14 | -0.16 | -0.15 | -0.19 | 0.14 | -0.20 | -0.17 | 0.24 | **-0.40** | 0.15 | -0.03 | 0.14 | 0.15 | 0.19 | 0.02 | **0.35** | **0.81** | **0.51** | **0.41** | 0.23 |  | **0.67** | 0.33 |
| Mg µg/mg DM | 0.24 | 0.21 | 0.01 | **-0.40** | -0.23 | -0.14 | -0.18 | -0.17 | **-0.50** | -0.12 | -0.14 | -0.16 | 0.06 | 0.06 | **-0.52** | 0.08 | -0.15 | 0.09 | 0.20 | -0.14 | **-0.34** | **0.47** | **0.53** | **0.81** | **0.35** | **0.43** | **0.53** |  | 0.19 |
| Ca µg/mg DM | **0.35** | 0.24 | 0.11 | **-0.44** | 0.01 | -0.14 | **-0.36** | -0.13 | -0.32 | -0.27 | 0.13 | **-0.41** | -0.33 | **0.38** | **-0.35** | **0.36** | 0.27 | -0.09 | 0.20 | **0.35** | 0.12 | **0.41** | **0.40** | **0.38** | **0.90** | **0.37** | 0.33 | 0.25 |  |

Correlations below the diagonal are for all 34 genetic lines; those above the diagonal are for 22 layer and traditional breeds; correlations in bold are significant at *p*<0.05

^1^Plasma creatine kinase activity
